# An Arabidopsis FANCJ helicase homologue is required for DNA crosslink repair and rDNA repeat stability

**DOI:** 10.1371/journal.pgen.1008174

**Published:** 2019-05-23

**Authors:** Annika Dorn, Laura Feller, Dominique Castri, Sarah Röhrig, Janina Enderle, Natalie J. Herrmann, Astrid Block-Schmidt, Oliver Trapp, Laura Köhler, Holger Puchta

**Affiliations:** Botanical Institute, Molecular Biology and Biochemistry, Karlsruhe Institute of Technology, Karlsruhe, Germany; Weizmann Institute, ISRAEL

## Abstract

Proteins of the Fanconi Anemia (FA) complementation group are required for crosslink (CL) repair in humans and their loss leads to severe pathological phenotypes. Here we characterize a homolog of the Fe-S cluster helicase FANCJ in the model plant Arabidopsis, AtFANCJB, and show that it is involved in interstrand CL repair. It acts at a presumably early step in concert with the nuclease FAN1 but independently of the nuclease AtMUS81, and is epistatic to both error-prone and error-free post-replicative repair in Arabidopsis. The simultaneous knock out of FANCJB and the Fe-S cluster helicase RTEL1 leads to induced cell death in root meristems, indicating an important role of the enzymes in replicative DNA repair. Surprisingly, we found that AtFANCJB is involved in safeguarding rDNA stability in plants. In the absence of AtRTEL1 and AtFANCJB, we detected a synergetic reduction to about one third of the original number of 45S rDNA copies. It is tempting to speculate that the detected rDNA instability might be due to deficiencies in G-quadruplex structure resolution and might thus contribute to pathological phenotypes of certain human genetic diseases.

## Author summary

The Fanconi Anemia and Hoyeraal-Hreidarsson syndromes are severe human genetic diseases that are correlated with DNA repair deficiencies. Interestingly, plants harbour homologues of factors in their genome that are causative for the respective diseases upon mutation. Using the model plant Arabidopsis, we analysed in detail the role of two helicases that are causing one or the other syndrome when defective in humans. We found that the simultaneous loss of both helicases leads to synergistic defects of plant cells in response to induced but also natural DNA damage, indicating that they act in different repair pathways. Most surprisingly, we found a drastic reduction of rDNA copies, in the double mutant, that are required for ribosome formation and thus protein synthesis. Such a phenotype has not been reported before—neither for humans nor animals—but this finding might significantly contribute to specificities of the respective diseases.

## Introduction

The helicase FANCJ was initially identified in humans as a direct interaction partner of the tumour suppressor protein BRCA1, thereby originally being named BACH1 (BRCA1-associated C-terminal helicase 1) or BRIP1 (BRCA1 interacting protein 1) [1]. The helicase was later determined as the causal gene of the Fanconi Anemia complementation group J [2–4]. Fanconi Anemia (FA) is a rare human hereditary disease, characterized by bone marrow failure and a high cancer predisposition [5]. At least 22 genes were identified in causing FA upon mutation, including the two helicases FANCM and FANCJ [6]. On a cellular level, FA patients exhibit a characteristic hypersensitivity against DNA crosslinking agents such as Mitomycin C (MMC), hinting to the important function of the FA proteins in interstrand-crosslink (ICL) repair [7]. ICLs pose a serious threat to genome stability as they connect both strands of the DNA double helix, thereby blocking replication and transcription. The FANC genes cooperate in the human canonical ICL repair pathway. Its exact repair mechanism is still under investigation, but damage recognition relies on the helicase FANCM. FANCM, together with at least eight further FA proteins, forms the FA core complex that initiates the monoubiquitination of the FANCD2/I heterodimer. This leads to the recruitment of a multitude of ICL repair factors, catalysing the nucleolytic excision of the crosslink (unhooking), resulting in a one-sided double strand break (DSB). The DSB is repaired via homologous recombination, while translesion synthesis (TLS) bypasses the unhooked crosslink, which can be removed by further processing by nucleotide excision repair (NER) factors [5]. The FA factor FANCJ is a Fe-S cluster helicase with 5’-3’ directionality, acting on dsDNA substrates with 5’ ssDNA overhangs, but also unwinds D-Loops without single-stranded regions [8,9]. The exact function of FANCJ in the FA pathway is still obscure, but it was shown that the helicase is not necessary for FANCD2 monoubiquitination, indicating a possible later involvement [4]. FANCJ was demonstrated to act together with its interaction partner BRCA1 in promoting HR following DSBs, however FANCJ also has a destabilizing function on RAD51 nucleoprotein filaments [10,11]. Furthermore, a role for FANCJ besides from the FA pathway, has been postulated with a function in the removal of G-quadruplex (G4) DNA structures[12,13]. Interestingly, a direct interaction of FANCJ and the RecQ helicase BLM was shown, and both helicases seem to cooperate in response to replicative stress [14,15]. These various functions hint to a multifunctional role of FANCJ in the maintenance of genome stability. In the plant model organism *Arabidopsis thaliana*, only a small subset of FA factors could be identified so far, with the helicase FANCM being the only plant FA protein involved in CL repair [16]. However, AtFANCD2 and AtFANCM have been shown to be involved in meiotic recombination [16–18]. Nevertheless, CL repair functions could be described for Arabidopsis homologs of the FA-associated proteins FAN1 and MHF1 [19,20]. Although two Arabidopsis FANCJ paralogs had been identified by our group several years ago, further characterization had not been carried out till now [21].

Closely related to FANCJ is a further Fe-S cluster helicase, RTEL1, for which an involvement in G4 DNA metabolism was also reported [22]. Mutations in RTEL1 lead to Hoyeraal-Hreidarsson syndrome in humans, a severe form of dyskeratosis congenital that is characterized by bone marrow failure, immunodeficiency and growth retardation [23–26]. Mammalian RTEL1 was shown to act in the preservation of telomere stability, as it dissolves T-Loops and telomeric G4 DNA structures, furthermore loss-of-function mutations lead to telomere-loss and chromosome fusions [22,27]. Thereby a parallel involvement of RTEL1 and the RecQ helicase BLM could be shown. In *C*. *elegans*, a role for RTEL1 in ICL repair was demonstrated, furthermore the helicase was characterized as a antirecombinase that suppresses recombination by disassembling D-Loops [28]. Interestingly, simultaneous loss of RTEL1 and the FANCJ homolog here lead to synthetic lethality, indicating independent essential roles for both helicases [28]. For the Arabidopsis RTEL1 homolog, its antirecombinogenic function, as well as its involvement in CL repair, was demonstrated [21,29]. The RTEL1 mediated suppression of HR defines a pathway independent of the FA helicase FANCM or the RTR complex [21,30]. AtRTEL1 seems to fulfil multifaceted functions in stabilizing the genome, with a role in telomere homeostasis but also a role in the maintenance of 45S rDNA repeat number parallel to the RTR complex being shown [21,30]. This highlights the importance of RTEL1 in preserving repetitive sequences within the plant genome.

As the classical FA pathway for ICL repair does not seem to be conserved in plants, we set out to characterize their specific network of crosslink repair factors over the last recent years. Initially, a three-branched model of CL repair, defined by the RecQ helicase RECQ4A, the nuclease MUS81 and the ATPase RAD5A, has been proposed [31]. AtRECQ4A is the functional homolog of the human BLM helicase and part of the evolutionary conserved RTR complex [32,33]. RECQ4A was shown to act in ICL repair in a common pathway with the FA-associated nuclease FAN1 but independently of FANCM [19,20]. The ATPase AtRAD5A is the functional homolog of yeast Rad5 and acts in the error-free branch of post-replicative repair (PRR). For the Arabidopsis homolog, a function in DNA repair, apart from other pathways such as nucleotide excision repair, single-strand break repair, micro-homology mediated double-strand break repair and the ATM-mediated DNA damage response, was demonstrated [34–36]. The endonuclease MUS81 seems to mediate a separate backup pathway for the resolution of repair intermediates, but interestingly acts in a common pathway with the helicase RTEL1 [21]. Furthermore, a parallel involvement of MUS81 and FAN1 in CL repair was shown. As double mutants of *MUS81* with the RTR complex partners *RECQ4A* and *TOP3α* or the FA helicase *FANCM* exhibit synthetic lethality, this further emphasizes the importance of these parallel pathways in the repair of replicative DNA damage [20,37,38]. MUS81 was only recently identified as the central player in DNA-protein crosslink repair, where the nuclease acts in parallel to the protease WSS1A and the tyrosyl-DNA-phosphodiesterase TDP1 [39]. REV3, the catalytic subunit of the translesion synthesis (TLS) DNA polymerase Zeta is an additional important player in ICL repair, as TLS acts downstream of the FA pathway and in the error-prone post-replicative repair [5]. Mutants for the Arabidopsis homolog At*REV3* were also shown to be sensitive towards MMC induced DNA damage, indicating conserved functions [40].

We now wanted to characterize the role of FANCJ homologs from Arabidopsis in ICL repair for the first time, thereby aiming to include them into the CL repair network. Surprisingly, only one FANCJ homolog, AtFANCJB, turned out to possess a function in ICL repair, but also, an exciting role of this homolog in the maintenance of 45S rDNA repeat stability in parallel to the related helicase RTEL1 could be revealed.

## Results

### AtFANCJB is involved in ICL repair but not in the suppression of HR

**Fig 1:**
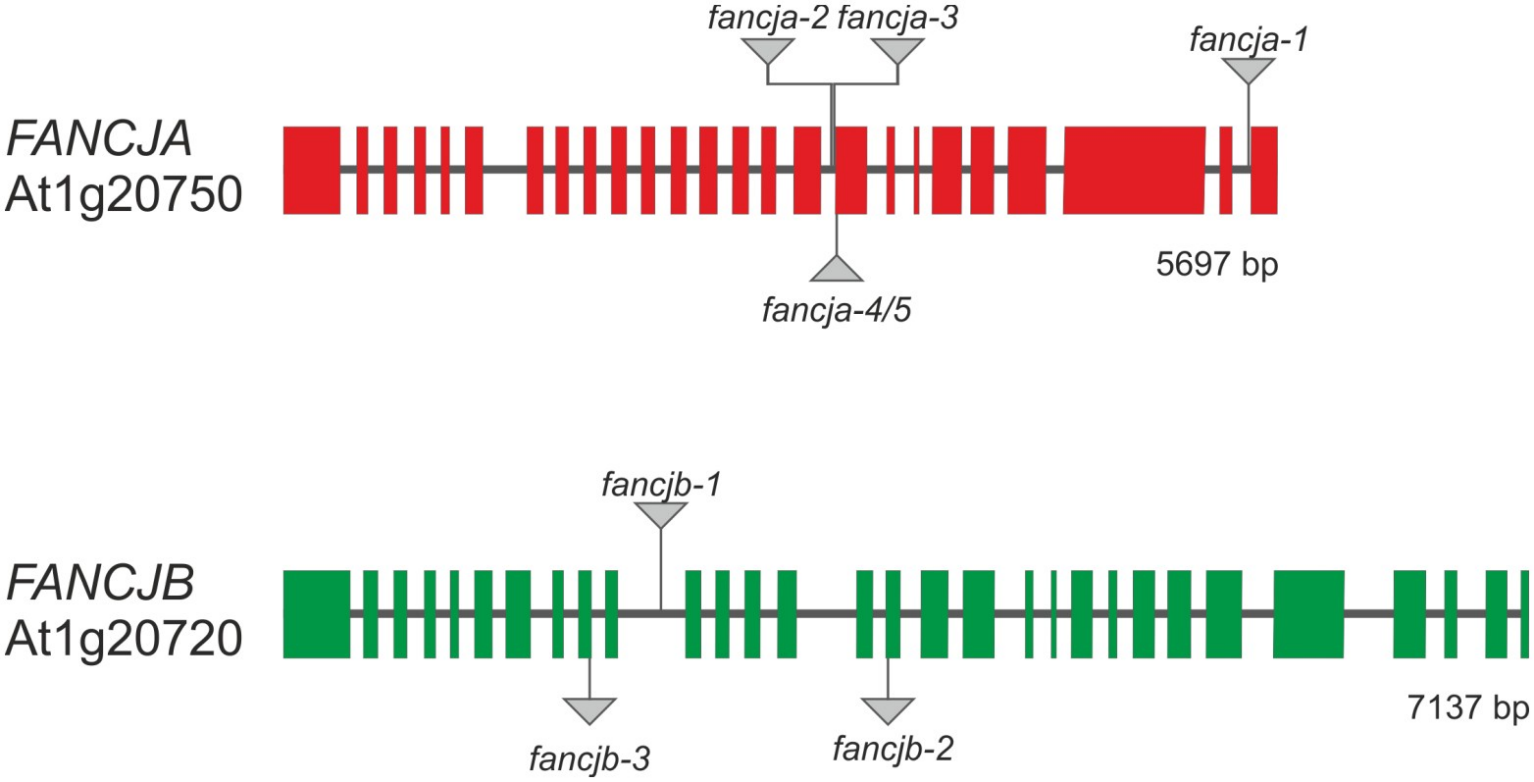
Gene structure of *FANCJ* homologs in *Arabidopsis thaliana*. At*FANCJA* is located at the locus At1g20750 and comprises of 25 exons (red boxes) and 24 introns (black bars) in 5697 bp. The T-DNA insertion in *fancja-1* is integrated in intron 24. The mutations in *fancja-2/3* are located spanning intron 16/exon 17, the insertions in *fancja-4/5* are inserted in exon 17. At*FANCJB* (At1g20720) consists of 7137 bp in 30 exons (green boxes) and 29 introns (black bars). In *fancjb-1*, a T-DNA is inserted in intron 10, *fancjb-2* harbours a deletion in exon 16 and *fancjb-3* contains a deletion in exon 9.

Through BLAST analyses with the human FANCJ protein sequence, two FANCJ homologs could be identified in the Arabidopsis genome, named FANCJA (At1g20750) and FANCJB (At1g20720). The AtFANCJ proteins are 27.5% and 27.4% identical to HsFANCJ, respectively and 66.2% identical to each other. At*FANCJA* comprises of 5697 bp with 25 exons and 24 introns, while At*FANCJB* harbours 30 exons and 29 introns in 7137 bp (Fig 1). Only two genes on chromosome 1 thereby separate the genes from each other. We analysed expression profiles for both genes based on the TraVA database [41]. Thereby *FANCJA* exhibited an extremely low expression, with the highest expression level found in anthers. However, the expression level of *FANCJB* was at least one order of magnitude higher, with the highest expression found in meristems. For the functional characterization of these FANCJ homologs in Arabidopsis, one T-DNA insertion line for each gene was analysed. In *fancja-1* (SALK_079991) the T-DNA insertion is located in intron 24, while the T-DNA in *fancjb-1* (SALK_101493) is inserted in intron 10 and associated with a genomic deletion of 15 bp (S1 Fig). Additionally, we generated further mutants for *FANCJA* and *FANCJB* via Cas9-mediated mutagenesis. The mutagenesis of At*FANCJA* was performed using a double nickase approach with *S*. *pyogenes* Cas9, as previously described [42]. Therefore, protospacers in intron 16 (5’-GAATAAGAATGCATCAGTTT-3’) and spanning intron 16/exon 17 (5’-AGAACCAAGAGGAGGATCCA-3’) were used. With this approach, two additional *fancja* mutant lines could be generated, termed *fancja-2* and *fancja-3* (S2 Fig). For the mutagenesis of At*FANCJB*, two independent *S*. *aureus* Cas9 nuclease approaches were performed [43]. The target sequences were located spanning exon 9/intron 10 (5’-TTACCTCTACTACTTCACAC-3’) and spanning intron 15/exon 16 (5’-TATCTTTGTTCCAGGTGTGG-3’). With each approach, one mutant line was obtained and named *fancjb-2* and *fancjb-3*, respectively (S4 Fig). The mutations in all produced lines lead to frameshifts within the open reading frame of the corresponding gene and thereby generated premature stop codons, most likely leading to an abortion of translation (S3 and S4 Figs). The *fancja* and *fancjb* mutant lines were indistinguishable from WT plants with regards to their growth phenotype, and were fully fertile (S5 Fig).

**Fig 2:**
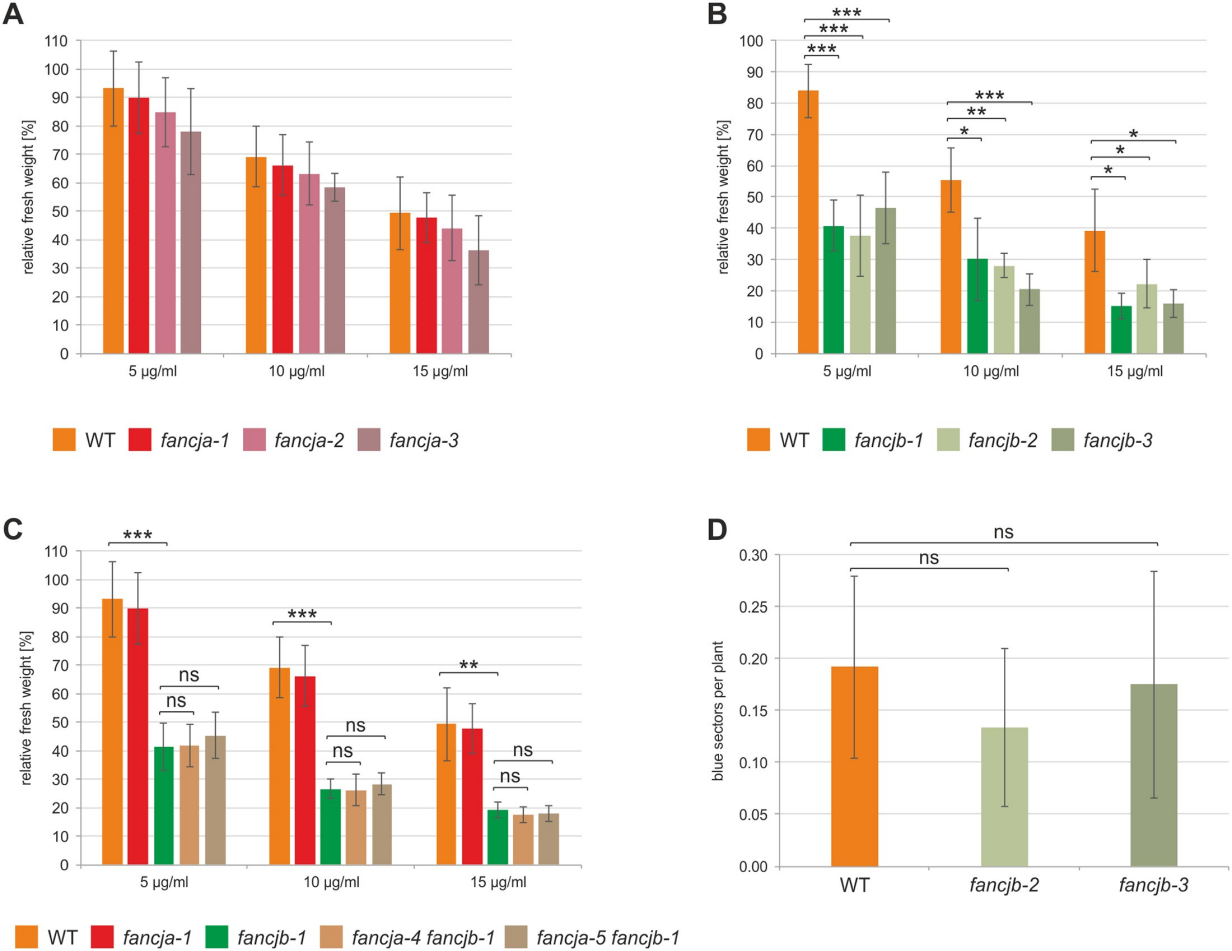
Sensitivity analysis of *fancja* and *fancjb* mutant lines after MMC treatment and HR analysis in *fancjb* mutant lines. The fresh weight of the individual lines in response to MMC treatment was determined in relation to untreated control plants. Five independent assays were performed and mean values with standard deviations (error bars) were calculated. (A) The relative fresh weight of all *fancja* mutant lines did not differ significantly from WT plants. (B) All three *fancjb* mutant lines depicted a significantly reduced fresh weight compared to WT plants at 5, 10 and 15 μg/ml MMC concentrations. (C) Both *fancja fancjb* double mutant lines were hypersensitive against MMC treatment, on a level comparable to the *fancjb* single mutant. (D) Homologous recombination frequency was determined with the IC9C reporter in three independent assays and mean values with standard deviations (error bars) were calculated. The number of blue sectors per plant in both *fancjb* mutant lines did not differ from WT plants. Statistical differences were calculated using the two-tailed t-test with unequal variances: * p < 0.05, ** p < 0.01, *** p < 0.001, ns = not significant.

As human FANCJ is a known factor involved in the FA pathway, we were interested in the function of the Arabidopsis FANCJ homologs in ICL repair. To test this, we analysed the obtained *fancja* and *fancjb* T-DNA and CRISPR/Cas9 mutant lines on their sensitivity towards the ICL inducing agent Mitomycin C (MMC). Therefore, we treated one-week-old plantlets of the individual lines with different MMC concentrations, incubated them for an additional two weeks and measured the resulting fresh weight. The fresh weight of treated plants was normalized to the fresh weight of untreated plants of the same line, to exclude the influence of growth differences. As a control, wild-type (WT) plants were treated accordingly. For all three tested *fancja* mutant lines, no significant differences in fresh weight compared to WT plants could be observed following MMC treatment (Fig 2A). In contrast to this, the three *fancjb* mutant lines exhibited a comparable hypersensitivity after treatment with 5, 10 and 15 μg/ml MMC, resulting in a significantly reduced fresh weight compared to WT plants (Fig 2B). This indicates an involvement of AtFANCJB in ICL repair. As mutants from other helicases such as At*RECQ4A*, At*FANCM* and At*RTEL1* show sensitivity in addition to a hyperrecombination phenotype, we were wondering whether the same holds true for At*FANCJB*. Therefore, we crossed the *fancjb-2* and *fancjb-3* mutant lines with the IC9C reporter line [44]. This line harbours an interrupted *GUS* gene that enables the quantification of interchromosomal recombination events upon restoration. However, both *fancjb* mutants tested did not differ in their recombination frequencies from that of WT plants (Fig 2D). Thus, AtFANCJB is not involved in the suppression of HR in plants.

### AtFANCJA is not involved in ICL repair

Although no direct function for FANCJA in ICL repair could be found, the homolog could still perform a possible hidden role, with a backup function for FANCJB. To address this question, we aimed to analyse *fancja fancjb* double mutants. As the two genes are both located on chromosome 1, separated by only 10 kb, the utilisation of crossbreeding for double mutant generation was not possible. To circumvent this problem, we performed a Cas9-mediated mutagenesis of *FANCJA* in the *fancjb-1* mutant line. Therefore, we used the same double nickase approach as for the *fancja* single mutant generation. Two individual mutant lines for *FANCJA* could be obtained in the *fancjb-1* mutant background, resulting in the double mutants *fancja-4 fancjb-1* and *fancja-5 fancjb-1*. The *FANCJA* mutations in both double mutants lead to a frameshift in the open reading frame of the *FANCJA* gene, thus resulting in a premature stop codon (S3 Fig). We tested the obtained double mutants on their sensitivity towards MMC in comparison to the *fancja-1* and *fancjb-1* single mutants and WT plants. While *fancja-1* mutants did not display MMC hypersensitivity, *fancjb-1* mutants showed a significantly reduced fresh weight in comparison to the WT (Fig 2C). Interestingly, both *fancja fancjb* double mutant lines exhibited hypersensitivity towards MMC on a level comparable to the *fancjb-1* single mutant, in all tested concentrations. Thus, AtFANCJA does not contribute to ICL repair and AtFANCJB seems to be the only functional FANCJ homolog in Arabidopsis.

### AtFANCJB acts in a common ICL repair pathway with the nuclease AtFAN1 and the helicase AtRECQ4A

**Fig 3:**
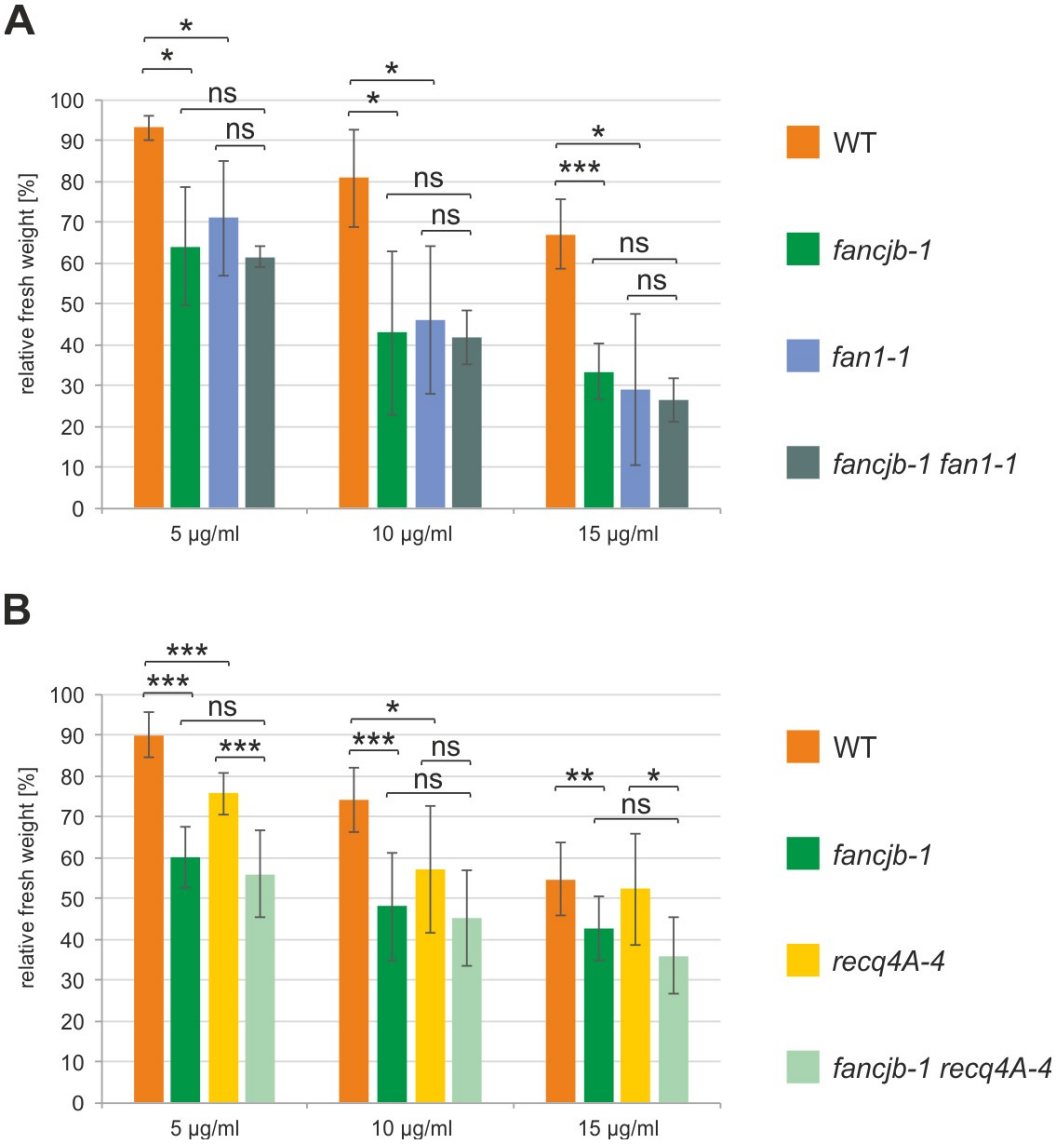
Sensitivity analysis of *fancjb-1 fan1-1* and *fancjb-1 recq4A-4* double mutants in response to MMC treatment. Relative fresh weight of double mutants, the respective single mutants and wild-type plants (WT) was determined after MMC treatment. Independent assays were performed at least 4 times and mean values with standard deviations (error bars) were calculated. (A) The relative fresh weight of *fancjb-1 fan1-1* double mutants was comparable to both single mutant lines in all tested MMC concentrations. (B) The *fancjb-1 recq4A-4* double mutants exhibited a relative fresh weight that did not significantly differ from *fancjb-1* single mutants. Statistical differences were calculated using the two-tailed t-test with unequal variances: * p < 0.05, ** p < 0.01, *** p < 0.001, ns = not significant.

As we were able to demonstrate a role for AtFANCJB in ICL repair, we wanted to define in detail as to which branch of the quite complex ICL repair network of plants it is acting in. Therefore, we generated double mutants with known ICL repair factors, by crossbreeding of the corresponding single mutants with *fancjb-1*, and analysed them in sensitivity assays with MMC. The nuclease FAN1 belongs to a group of FA-associated factors that are necessary for ICL repair in humans, but not causal for FA upon mutation [5,45]. FAN1 was postulated to be involved in the initial unhooking step in ICL repair, where the nuclease likely cuts opposite from either MUS81-EME1 or XPF/ERCC1 [46]. Interestingly, FAN1 is not conserved in all eukaryotes, as functional homologs could be found in mammals and *S*. *pombe*, whilst Xenopus FAN1 does not seem to be involved in ICL repair and no homologs were found in Drosophila or *S*. *cerevisiae* [47,48]. For AtFAN1, a conserved function in ICL repair upstream of both the RecQ helicase RECQ4A and the ATPase RAD5A, but in parallel to the nuclease MUS81, could be demonstrated [19]. We were therefore now interested as to whether AtFANCJB, as functional homolog of a known FA factor, acts together with the FA-associated nuclease AtFAN1 in ICL repair. Thus, we tested the *fancjb-1 fan1-1* double mutant for its sensitivity against MMC (Fig 3A). Both *fancjb-1* and *fan1-1* single mutants exhibited a comparable hypersensitivity against all tested concentrations of MMC, reflecting the roles of FANCJB and FAN1 in ICL repair. The double mutant featured a reduced fresh weight compared to WT plants, but did not differ significantly from either single mutant. Thus, FANCJB and FAN1 seem to participate in a common pathway in ICL repair.

The RecQ helicase AtRECQ4A is the functional homolog of the human BLM helicase and possesses important functions in various DNA repair and recombination processes [33,49]. Although *recq4a* mutants in Arabidopsis do not exhibit strong MMC sensitivity, a role in ICL repair could be unveiled by double mutant analyses. Thereby, RECQ4A could be classified into a repair pathway parallel to the FA helicase FANCM [20]. Consequently, it was of special interest to classify the other FA helicase from Arabidopsis, FANCJB, into the ICL repair pathways with regard to its cooperation with the RecQ helicase RECQ4A. While the *recq4A-4* single mutant only showed a slightly reduced fresh weight compared to WT plants following MMC treatment with 5 and 10 μg/ml, the *fancjb-1 recq4A-4* double mutant was comparable to the *fancjb-1* single mutant in all tested MMC concentrations (Fig 3B). This hints to a joint involvement of both helicases in ICL repair.

### AtFANCJB cooperates with the PRR factors AtRAD5A and AtREV3 in ICL repair

**Fig 4:**
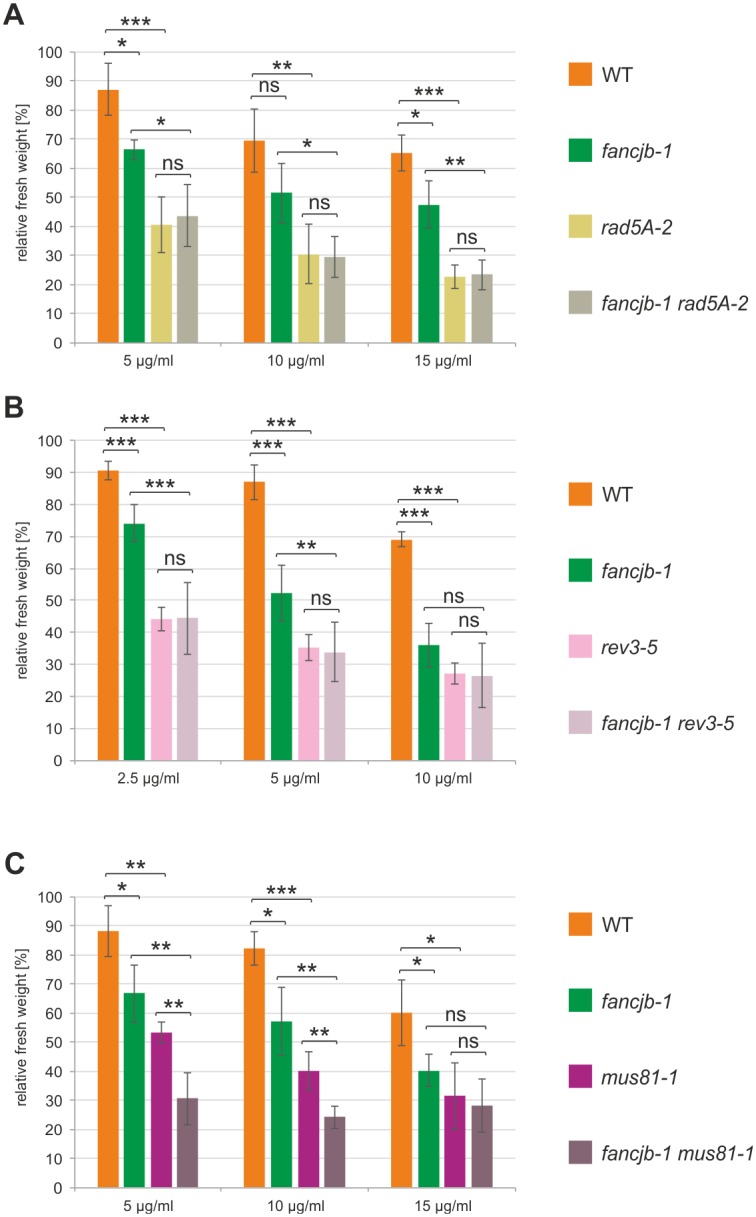
Sensitivity analysis of *fancjb-1 rad5A-2*, *fancjb-1 rev3-5* and *fancjb-1 mus81-1* double mutants in response to MMC treatment. Relative fresh weight of double mutants in comparison to the corresponding single mutants and wild-type plants (WT) was determined after MMC treatment. At least 4 independent assays were performed and mean values with standard deviations (error bars) were calculated. (A) The relative fresh weight of *fancjb-1 rad5A-2* double mutants was comparable to the *rad5A-2* single mutant in all tested MMC concentrations. (B) The *fancjb-1 rev3-5* double mutant exhibited a relative fresh weight that was not significantly different from the *rev3-5* single mutant after treatment with MMC. (C) In *fancjb-1 mus81-1* double mutants, a significantly reduced fresh weight compared to both single mutants could be observed after 5 and 10 μg/ml MMC treatment. Statistical differences were calculated using the two-tailed t-test with unequal variances: * p < 0.05, ** p < 0.01, *** p < 0.001, ns = not significant.

AtRAD5A was shown to be the functional homolog of the PRR factor ScRad5 and acts in the repair of DNA methylations and crosslinks [35]. Thereby, a function independent of NER, SSB repair and MMEJ could be demonstrated [34]. In ICL repair, RAD5A mediates a RECQ4A-independent sub-pathway downstream of the FA-associated nuclease FAN1 [19]. In the two-branched model of PRR in yeast, Rad5 is involved in the error-free pathway, both in the polyubiquitination of PCNA and mechanistically as a DNA translocase [50–52]. The error-prone pathway is mediated by translesion synthesis (TLS), where specialised polymerases are able to bypass DNA lesions [53]. REV3 is the catalytic subunit of the TLS polymerase Zeta, in which for the Arabidopsis homolog, an involvement in the repair of different DNA damages including ICLs was shown [40,54,55]. Furthermore, the parallel involvement of REV3 and RAD5A in DNA repair demonstrated the conservation of the two-branched PRR model in Arabidopsis [56]. For further analysis of the role of FANCJB in ICL repair, we analysed double mutants with the PRR factors *RAD5A* and *REV3* in response to MMC (Fig 4). At*rad5A-2* mutants exhibited a strong MMC sensitivity in all tested concentrations, while *fancjb-1* mutants depicted hypersensitivity after treatment with only 5 and 15 μg/ml (Fig 4A). For *fancjb-1 rad5A-2* double mutants, no further reduction in fresh weight could be detected, resulting in a fresh weight comparable to *rad5A-2* single mutants. For *rev3-5* mutants, hypersensitivity against all tested MMC concentrations could be observed (Fig 4B). The simultaneous mutation of *FANCJB* and *REV3* in the corresponding double mutant resulted in MMC hypersensitivity on a level comparable to the *rev3-5* single mutant in all tested concentrations. These results imply a cooperation of FANCJB with both RAD5A and REV3 in ICL repair, combining the error-free and error-prone branch of PRR into a common pathway with a FA factor in Arabidopsis.

### AtMUS81 and AtFANCJB act independently in ICL repair

The endonuclease AtMUS81 was shown to act on various branched DNA structures together with its complex partner EME1 and is thereby involved in the repair of a variety of DNA lesions [38,57]. AtMUS81 defines a special kind of backup pathway in ICL repair, where the nuclease acts separate from most other known factors [19,31]. For mammalian MUS81, an involvement parallel to FA signalling was demonstrated in ICL repair [58]. Interestingly, the simultaneous mutation of *MUS81* and the FA helicase *FANCM* in Arabidopsis leads to severe developmental defects, indicating independent roles in the repair of replicative DNA damage [18,20]. These findings raised questions of the functional relationship of MUS81 and FANCJB in ICL repair. Therefore, we analysed *fancjb-1 mus81-1* double mutants on their sensitivity towards MMC, as no growth defects were detectable in untreated plants. Both the *fancjb-1* and *mus81-1* single mutants exhibit a reduced fresh weight after treatment with 5, 10 and 15 μg/ml MMC, compared to WT plants (Fig 4C). In *fancjb-1 mus81-1* double mutants, an additive sensitivity could be observed at 5 and 10 μg/ml MMC. This led us to the conclusion that MUS81 mediates an independent pathway from FANCJB in ICL repair.

### The helicases AtRTEL1 and AtFANCJB are independently involved in ICL repair and replication-associated DNA repair in the root meristem

**Fig 5:**
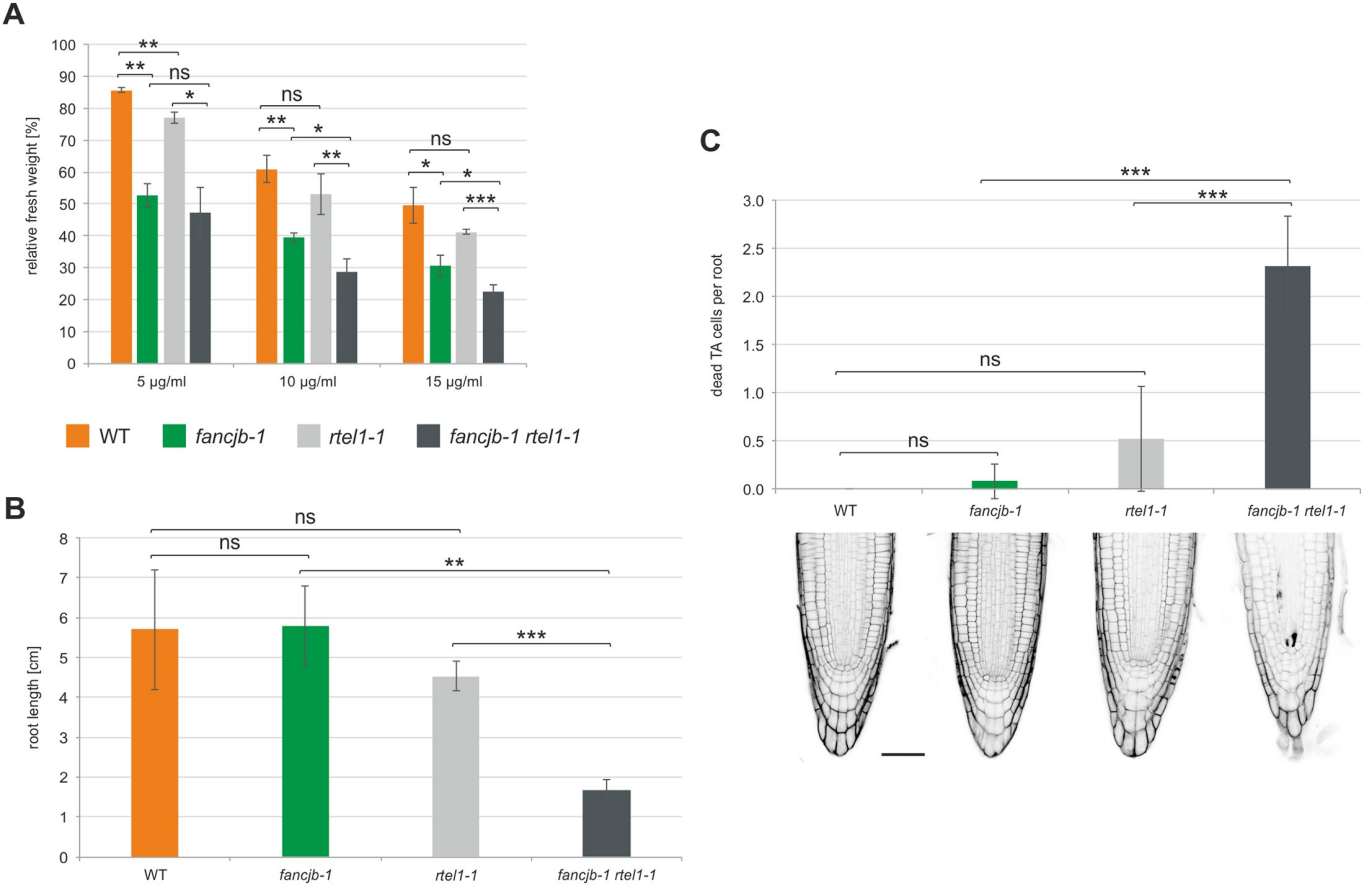
Epistasis analysis of FANCJB and RTEL1 in interstrand CL repair, root length and cell death analysis. (A) Relative fresh weight of *fancjb-1 rtel1-1* double mutants, the respective single mutants and wild-type plants (WT) was determined after MMC treatment (n = 3). The *fancjb-1 rtel1-1* double mutant depicted a significantly reduced fresh weight compared to both single mutants after treatment with 10 and 15 μg/ml MMC. (B) Root length of 10-day-old *fancjb-1 rtel1-1* double mutants was determined in comparison to the respective single mutants and WT plants (n = 4). The roots of *fancjb-1 rtel1-1* double mutants were significantly shorter than roots of either single mutant. (C) Cell death analysis in the root meristem of *fancjb-1 rtel1-1* double mutants in comparison to the corresponding single mutants and WT plants (n = 5, scale bar = 50 μm). In *fancjb-1 rtel1-1* double mutants, a significantly increased amount of dead transiently amplifying (TA) cells, in comparison to both single mutants, could be observed. Statistical differences were calculated using the two-tailed t-test with unequal variances: * p < 0.05, ** p < 0.01, *** p < 0.001, ns = not significant.

FANCJ and RTEL1 are closely related helicases that both belong to the XPD helicase family [59]. This helicase family encompasses a number of conserved proteins important for genome stability, and FANCJ and RTEL1 homologs are both present in animals and plants [21,60]. For mammalian RTEL1, important functions in telomere stability have been demonstrated, and RTEL1 was shown to be necessary for T-Loop dismantling during replication [22,27]. RTEL1 homologs exhibit important roles in ICL repair, as shown for *C*. *elegans* and human cell lines [28]. For Arabidopsis, a conserved function for RTEL1 in ICL repair could be demonstrated, where the helicase interestingly acts in a common pathway with the endonuclease MUS81, but in a pathway parallel to the helicases FANCM and RECQ4A [21]. Of course, this raised questions regarding the genetic interaction of RTEL1 and FANCJB in ICL repair. Therefore, we analysed *fancjb-1 rtel1-1* double mutants in sensitivity assays with MMC treatment (Fig 5A). For *rtel1-1* single mutants, only a slight hypersensitivity could be observed after treatment with 5 μg/ml MMC, while *fancjb-1* single mutants exhibited a strong reduction of fresh weight compared to WT plants at 5, 10 and 15 μg/ml MMC concentrations. In *fancjb-1 rtel1-1* double mutants a further reduction of fresh weight, compared to both single mutants, could be observed after treatment with 10 and 15 μg/ml MMC. Thus, FANCJB and RTEL1 are involved in independent pathways of ICL repair.

In Arabidopsis, RTEL1 was furthermore shown to be important for correct replication, as mutants depict a slow growth phenotype and shortened roots [21,29]. Thereby, a role for RTEL1 in the repair of replication-associated DNA damage in parallel to FANCM, MUS81 and RECQ4A, was demonstrated. As FANCJB is closely related to RTEL1, we were now interested in the function of both factors in replication-associated DNA repair. Spontaneously occurring DNA damage can lead to blockades of the replication machinery, therefore slowing cell division. As root growth in plants relies on cell divisions within the root meristem, it is a suitable system for documenting the impact of replication-associated DNA damage [61]. Therefore, we analysed the root length of 10-day-old *fancjb-1 rtel1-1* double mutants in comparison to the corresponding single mutants and WT plants (Fig 5B). While both single mutant lines exhibited a root length comparable to WT plants under these conditions, root length in the double mutant was significantly reduced, with a remaining length of less than a third of WT roots. We wanted to take a closer look at the roots and the possible cause of this phenotype, therefore we analysed cell death in the root meristem. This is based on the staining of roots from five-day-old seedlings with the nucleic acid intercalating dye, propidium iodide (PI). PI is only able to pass the perforated cell membrane of dead cells and thereby allows the differentiation between living and dead cells. The stem cell niche of the root meristem consists of stem cells that are grouped around the quiescent centre. Via the constant division of these cells, the different cell types in roots are formed. The transiently amplifying (TA) cells are located apical of the stem cell niche, that are characterized by frequent cell divisions and therefore are especially well suited for the analysis of replication-associated DNA repair [61]. Cells of the root meristem are extraordinarily sensitive against genotoxic stress and feature a selective occurrence of programmed cell death, even in response to a low level of DNA damage [62]. We analysed the number of dead TA cells in root tips of *fancjb-1 rtel1-1* double mutants in comparison to both single mutants and WT plants (Fig 5C). No dead TA cells were visible in WT roots and both single mutants did not depict a significantly different number of dead TA cells compared to WT roots. Consistent with the root length results, in *fancjb-1 rtel1-1* double mutants, an increased number of dead TA cells per root could be observed. Thus, the short root phenotype of *fancjb-1 rtel1-1* double mutants seems to be caused by an increased cell death in the root meristem. This is likely because of parallel functions of both helicases in the repair of replication-associated DNA damage.

### AtRTEL1 and AtFANCJB act in parallel pathways in the maintenance of rDNA repeat stability

**Fig 6:**
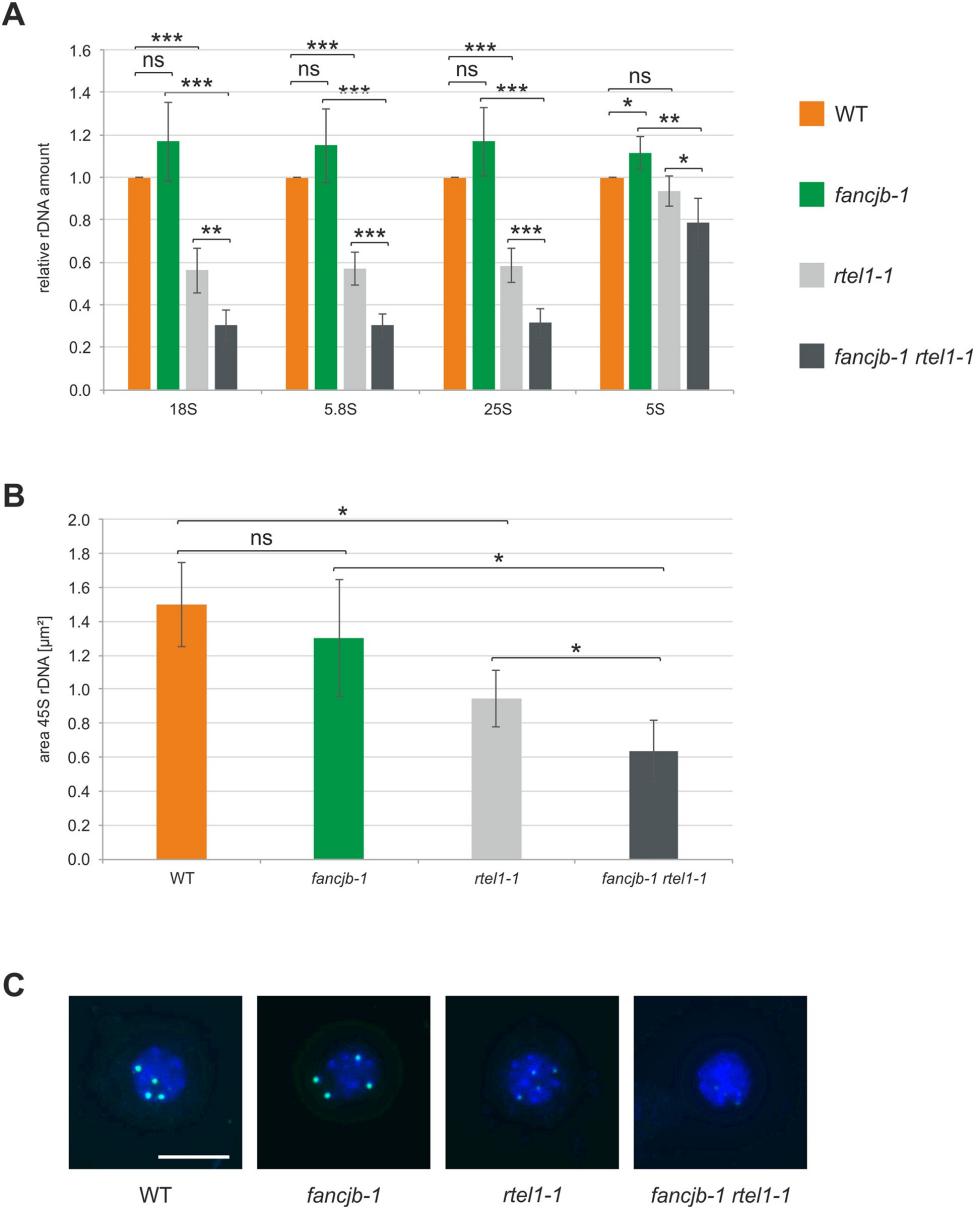
Analysis of rDNA repeat number in *fancjb-1 rtel1-1* double mutants. (A) The rDNA repeat amount was determined by quantitative real-time PCR in the *fancjb-1 rtel1-1* double mutant and corresponding single mutants, in relation to WT plants (n = 5). The 45S rDNA repeat number was determined using individual primer pairs for the 18S, 5.8S and 25S rDNA sequences. While the 45S rDNA copy number in the *fancjb-1* single mutant was comparable to WT plants, the *fancjb-1 rtel1-1* double mutant exhibited a copy number significantly reduced to both single mutants. The 5S rDNA repeat number also showed a significant reduction in the *fancjb-1 rtel1-1* double mutant compared to both single mutants. (B) The reduction in 45S rDNA amount was additionally quantified by FISH (fluorescence in situ hybridization), using an Atto488 labelled 45S rDNA probe and DAPI staining of chromatin (n = 4). The area of the 45S rDNA was significantly reduced in the *fancjb-1 rtel1-1* double mutant compared to both single mutants. (C) Exemplarily chosen nuclei from each genotype depicting chromatin (blue) with the four 45S rDNA loci (green, scale bar = 10 μm). Statistical differences were calculated using the two-tailed t-test with unequal variances: * p < 0.05, ** p < 0.01, *** p < 0.001, ns = not significant.

RTEL1 was shown to be an important factor for the stability of repetitive DNA sequences. The 45S rDNA loci on chromosome 2 and 4 build a large cluster of repetitive sequences in the Arabidopsis genome. In a previous study, we were able to demonstrate an important function for AtRTEL1 in stabilizing 45S rDNA repeats. 45S rDNA repeat number in *rtel1-1* mutants is reduced to about half of that of WT plants. Thereby, a parallel involvement of RTEL1 and the RTR complex partner RMI2 in 45S rDNA maintenance could be observed, as double mutants suffer from a further diminished 45S rDNA copy number to only a third of WT plants [30]. Human FANCJ was postulated to act in supressing microsatellite instability, in a function independent of the FA pathway [63,64]. This led us to question whether AtFANCJB might also be involved in rDNA maintenance. To determine this, we analysed the relative number of 45S and 5S rDNA repeats in two-week-old *fancjb-1* single and *fancjb-1 rtel1-1* double mutants (third homozygous generation), compared to *rtel1-1* mutants and WT plants, via quantitative real-time PCR (Fig 6A). We were able to reproduce the strong reduction of 18S, 5.8S and 25S rDNA amounts in *rtel1-1* single mutants, while no reduction in rDNA copy number could be observed in *fancjb-1* single mutants compared to WT plants. Astonishingly, in *fancjb-1 rtel1-1* double mutants, the amount of all three 45S rDNA coding sequences was significantly further reduced, compared to *rtel1-1* single mutants, to only about 30% of WT plants. Additionally, we included an analysis of 5S rDNA repeat number. The 5S rDNA loci, coding for the 5S rRNA, are located in the pericentromeric regions of chromosome 3, 4 and 5 in Arabidopsis [65,66]. Interestingly, 5S rDNA copy number in the *rtel1-1* single mutant did not differ from WT copy number and *fancjb-1* single mutants even depicted a slight increase in the amount of 5S rDNA. In contrast to this, the *fancjb-1 rtel1-1* double mutant possessed a significantly reduced copy number of about 80% of WT plants. To confirm the results concerning the 45S rDNA with a cytological method, visualizing the reduction in rDNA copy number, we analysed the *fancjb-1 rtel1-1* double mutant, the corresponding single mutants and WT plants in a FISH (fluorescence in situ hybridisation) analysis (Fig 6B and 6C). Therefore, we used a 45S rDNA probe labelled with Atto488 and DAPI staining in chromatin spreads, as described before [30]. With this method, we were able to confirm the drastic reduction in 45S rDNA amount in *fancjb-1 rtel1-1* double mutants compared to both single mutant lines. These results indicate an important function for FANCJB in the maintenance of the 45S rDNA repeats in parallel to RTEL1. Furthermore, both helicases are likely to also be involved in the stabilisation of 5S rDNA repeats. This further underlines a key function for FANCJB in maintaining genome stability in Arabidopsis, not only in ICL repair, but also in the homeostasis of repetitive sequences.

## Discussion

The human FANCJ helicase is involved in the Fanconi Anemia (FA) pathway of ICL repair and mutations in the corresponding gene lead to the eponymous hereditary disease FA [2–4]. In this study, we were able to identify FANCJB from *Arabidopsis thaliana* as the functional homolog of FANCJ in ICL repair. Furthermore, we were able to classify FANCJB into the ICL repair pathways of Arabidopsis and found a surprising involvement of FANCJB in the maintenance of rDNA repeat number in parallel to the related helicase RTEL1.

### FANCJB is the functional Arabidopsis FANCJ homolog in ICL repair

With bioinformatical analyses, we were able to identify two FANCJ homologs in the *Arabidopsis thaliana* genome, *FANCJA* and *FANCJB*. While both proteins share a high identity in amino acid composition, we could demonstrate a functional divergence. FANCJB is involved in ICL repair, apparent by the hypersensitivity of three independent *fancjb* mutant lines against MMC treatment. This is in accordance with the function of human FANCJ in the FA pathway of ICL repair, but also for FANCJ homologs in other animal systems [2–4,67,68]. As no such role was apparent for AtFANCJA, we further investigated a possible function of this homolog. The power of the CRISPR/Cas9 technology enabled us to generate double mutants [69]. A genetic approach for combining both mutant alleles was excluded as the genes are separated by only 10 kb on chromosome 1. Upon analysing the double mutant, we did not find any backup role for FANCJA in ICL repair. Thus, the function of FANCJA still remains elusive. It is noticeable that the expression level of FANCJA is at least one order of magnitude lower than the expression of FANCJB, and in general is hardly detectable [41]. This led us to the conclusion that FANCJB is the only functional Arabidopsis FANCJ homolog in ICL repair. As we were able to characterize a FANCJ homolog in a non-animal system for the first time, our results demonstrate that the role of FANCJ seems to be evolutionary conserved between animals and plants.

### Defining the role of FANCJB in the ICL repair pathway network of Arabidopsis

As we were able to identify FANCJB as functional FANCJ homolog in ICL repair, we were further interested in defining the role of FANCJB within the different repair pathways of Arabidopsis. Past analyses from our group defined a complex ICL repair network, with the already characterized factors FAN1, RECQ4A, RAD5A, REV3, MUS81 and RTEL1. With double mutant analyses, we now wanted to clarify if FANCJB acts in common or separate pathways with these proteins.

The nuclease FAN1 is a FA-associated factor that is essential for ICL repair in humans [45]. For the Arabidopsis homolog, an involvement in ICL repair has been demonstrated, where the nuclease acts upstream of two sub-pathways mediated by the RecQ helicase RECQ4A and the PRR ATPase RAD5A [19]. Our results now indicate a common involvement of FAN1 with the FA helicase FANCJB. As FAN1 is not conserved in all eukaryotes, its involvement in ICL repair seems to be also diverse in the different organisms where it is present. For chicken DT40 cells, a synergistically increased number of MMC-induced chromosomal aberrations could be determined in cells lacking both FAN1 and FANCJ [70]. Thus, there are clear differences in animals and plants in whether or not these two proteins act in a common repair pathway. It is tempting to speculate that in Arabidopsis both proteins cooperate with their complementing biochemical activities to process cross-linked DNA, to set it up for downstream repair. The nuclease might be required for breaking the DNA close to the CL, whereas the helicase might use the broken end to unwind the DNA at the damaged site.

AtRECQ4A is the functional homolog of the human BLM and the yeast Sgs1 helicase [33,49]. Studies from our group identified RECQ4A as crucial CL repair factor, defining a parallel pathway to RAD5A and MUS81 [31]. We were now able to show a common involvement of RECQ4A and FANCJB in ICL repair. For human FANCJ and BLM, a direct interaction could be demonstrated and the cellular level of BLM was shown to be dependent on FANCJ. Furthermore, BLM and FANCJ partially co-localize after MMC treatment of human cells [15]. This is in accordance with an involvement of BLM in the FA pathway, where additionally a function of BLM in the activation of FANCD2 was revealed [71]. Thus, the cooperation of both helicases in a common pathway seems to be evolutionary conserved. After the initial unwinding of the damaged DNA by FANCJB, RECQ4A might be involved in the further processing of these intermediates. These intermediates might then be processed by homologous recombination.

Post-replicative repair (PRR) is an important mechanism for the bypass of a wide variety of replication-blocking DNA damages, including ICLs. Thereby, PRR splits into an error-free and an error-prone branch, dependent on the ubiquitination state of PCNA. In yeast, the E3 ubiquitin ligase Rad5 was identified as an important positive regulator of the error-free PRR branch, with an additional mechanistical function as DNA translocase [50]. The error-prone pathway relies on the function of TLS polymerases that mediate replication past lesions, thereby potentially inducing mutations due to the lacking proofreading activity [53]. The two-branched PRR model is represented in *Arabidopsis thaliana* by the Rad5 homolog RAD5A and the TLS polymerase subunit REV3, that are both involved in ICL repair [35,40]. Our findings now suggest a common involvement of both PRR factors, together with FANCJB, in ICL repair, possibly with FANCJB acting upstream of the two separate PRR pathways. For Mph1, the yeast FANCM homolog, an epistatic interaction with Rad5-mediated DNA damage bypass was postulated [72]. This is especially interesting as the FA-pathway is not conserved in yeast, similar to Arabidopsis. In contrast to this, the human Rad5 homolog HLTF counteracts FANCJ in the replication stress response, indicating different circumstances in a situation with the presence of the FA pathway [73]. Also the interaction of error-prone PRR and FA-factors differs between organisms, as a common involvement of REV3 and FANCC in CL repair in DT40 cells was demonstrated, while human FANCJ enhances TLS after ICL induction [74,75]. For Arabidopsis, it seems that after the initial processing of the CL intermediates by FAN1 and FANCJB, in a sub-pathway, post-replicative repair factors can take over the processing of these intermediates as an alternative to the processing by RECQ4A.

FANCJ is ranked in a subclass of Fe-S helicases, including the helicases XPD, CHL1 and RTEL1 [59]. The involvement of AtRTEL1 in ICL repair was demonstrated before and the genetic interaction of the closely related helicase with FANCJB was of central interest in our study [21]. As *rtel1* mutants only depict a minor hypersensitivity after MMC treatment, FANCJB seems to fulfil a more important function in ICL repair. Nevertheless, as we found a synergistic hypersensitivity in *fancjb rtel1* double mutants, this implies independent roles for both helicases.

We further analysed the genetic interaction of FANCJB with the endonuclease MUS81. In Arabidopsis, MUS81 was postulated to define an important backup pathway for maintaining genome stability, as double mutants with a wide variety of DNA repair factors exhibit increased genotoxin sensitivity, severe growth retardation or synthetic lethality [31,37–39]. Our results clearly show that FANCJB is not processing CLs in a common pathway with MUS81 in plants. For mammalian systems, MUS81 was also demonstrated to contribute to genome stability, independently of the FA pathway. FANCD2 monoubiquitination following ICL induction was shown to be independent of MUS81, and ICL repair relies on the action of both FA-factors and MUS81 [58,76]. So in principle, two independent ways to initiate CL repair exist in plants [19].

**Fig 7:**
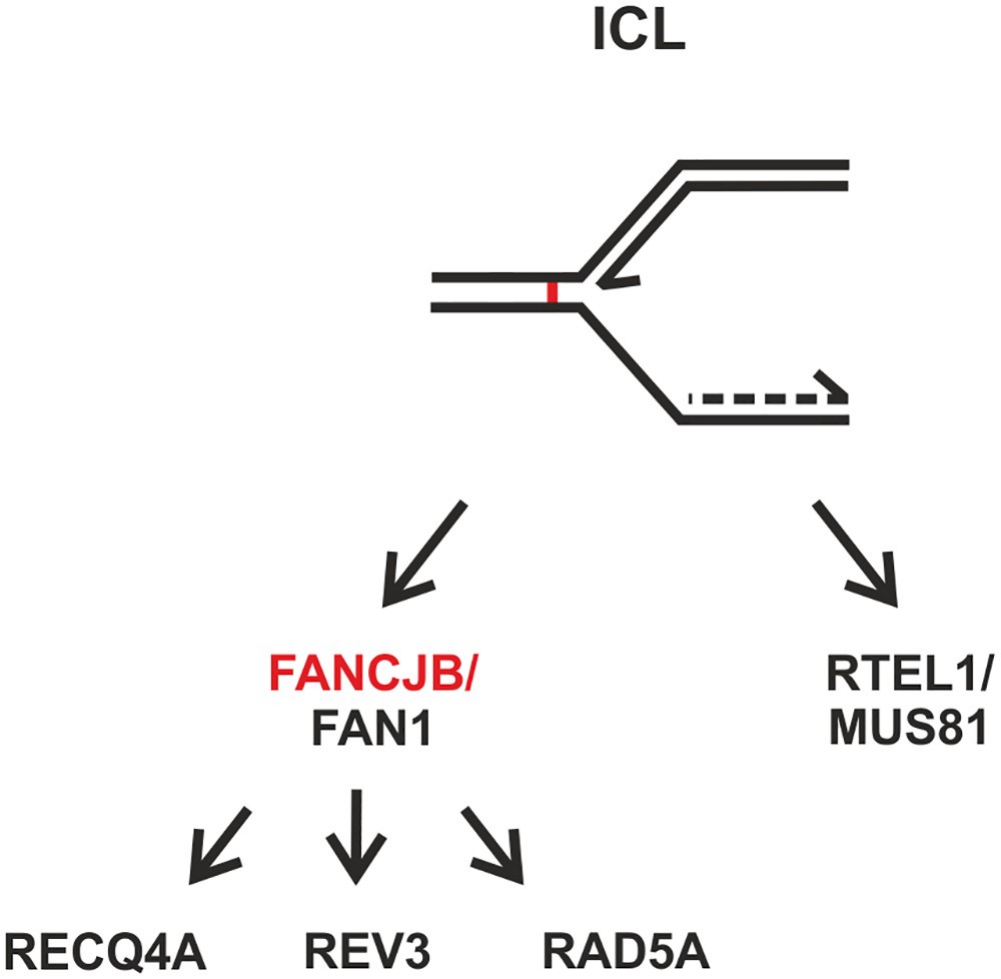
Classification of FANCJB into the interstrand CL repair network of *Arabidopsis thaliana*. FANCJB acts together with FAN1 in interstrand CL (ICL) repair, most likely upstream of three subpathways defined by RECQ4A, REV3 and RAD5A. FANCJB and MUS81 act in independent pathways, whereby RTEL1 could be classified into a common pathway with MUS81, separate from FANCJB.

With these analyses, we were now able to include FANCJB into the ICL repair network of *Arabidopsis thaliana* (Fig 7). FANCJB and FAN1 act in a common pathway initiating the repair reaction. The resulting intermediates are then alternatively processed by three sub-branches, defined by the RecQ helicase RECQ4A, in parallel to both PRR factors REV3 and RAD5A. A pathway separate from FANCJB is mediated by the backup-endonuclease MUS81. In this ICL repair pathway, the helicase RTEL1, for which we could now also show independent functions from FANCJB, is involved. Obviously both independent pathways require a Fe-S cluster kind of helicase for unwinding complex CL-associated DNA structures.

### FANCJB and RTEL1 are independently important for the stabilization of rDNA repeat number

As both XPD-like helicases FANCJB and RTEL1 are present in Arabidopsis, and double mutants depict a remarkably viable phenotype, this not only enabled analyses of both factors in ICL repair, but also concerning further functions of the helicases in maintaining genome stability. Double mutants for *rtel1* and the *C*. *elegans* FANCJ homolog *dog-1* depict a synthetically lethal phenotype, emphasizing the essential roles of both factors [28]. For AtRTEL1, important functions in replication-associated DNA repair have been demonstrated, as mutants exhibit various cell proliferation defects including a prolonged S-phase [29]. Furthermore, an increased cell death in the root meristem of *rtel1* single mutants, and an even further enhanced effect in double mutants with various DNA repair factors, underlined the significance of this helicase for correct DNA replication [21,30]. Our results also indicate a function for FANCJB in replication-associated DNA repair, as double mutants with *rtel1* exhibited a drastic shortening of roots, accompanied with an increase of cell death in the root meristem compared to both single mutants. Thus, FANCJB and RTEL1 independently contribute to a correct replication in the root meristem.

RTEL1 was demonstrated to be involved in the maintenance of repetitive DNA sequences. Recently, an important role for AtRTEL1 in the processing of telomeric recombination intermediates, arising through a RAD51-dependent HR pathway in the absence of telomerase, has been demonstrated [77]. Interestingly, AtRTEL1 was also shown to be involved in the maintenance of 45S rDNA repeat stability. The 45S rDNA genes are arranged in tandem repeats on chromosomes 2 and 4 and form one of the largest repetitive elements in the Arabidopsis genome [78,79]. The stability of the rDNA gene clusters is of special importance as they code for the catalytic rRNA core of ribosomes, essential for protein biosynthesis in all organisms. Our group demonstrated that the mutation of *RTEL1* in Arabidopsis plants is associated with a dramatic loss of 45S rDNA repeats to only 50% of WT copies. Thereby, RTEL1 acts independently of the RTR complex partner RMI2, as double mutants depict an even further loss of 45S rDNA copies [30]. This essential role for RTEL1 has been recently confirmed in the moss *Physcomitrella patens* [80]. Our analysis now also indicates a role for FANCJB in rDNA maintenance, as double mutants with RTEL1 exhibit a drastic loss of 45S rDNA repeat number compared to either single mutant, resulting in a copy number of only 30% of WT plants. We extended our analysis to include the investigation of 5S rDNA repeat number. In *Arabidopsis thaliana*, the 5S rDNA repeats are physically separated from the 45S rDNA loci and are located at the pericentromeric regions of chromosomes 3, 4 and 5. In our analysis, the 5S rDNA repeats seem to be more stable, as the loss of either RTEL1 or FANCJB did not result in a decrease in copy number, only the joint mutation of both helicases leads to a reduced 5S rDNA repeat number to about 80%. This indicates differences for the stability of 5S and 45S rDNA loci in Arabidopsis, and further analyses of factors differentially involved in the stabilisation might shed light on the underlying mechanism. Previous studies support this finding, as the copy number variation of 45S and 5S rDNA repeats in different Arabidopsis ecotypes did not show any correlation [81]. Thus, the epigenome might play an important role as transcription of the two rDNA species is mediated by different RNA polymerases, and factors associated with chromatin were shown to influence 45S, but not 5S, rDNA repeat stability [82,83]. As a role in the maintenance of epigenetic stability at G4-prone DNA sequences was shown in animals for FANCJ, it is tempting to speculate on such a possible function in Arabidopsis as well [84]. A further factor might be the localization on different genomic loci, as the 5S and 45S rDNA repeats in *P*. *patens* are linked in a common arrangement and were shown to react with a similar copy number reduction after *RTEL1* mutation [80]. As we now uncovered this change in rDNA copy number, it would also be interesting to analyse these effects in a multi-dimensional approach in the future, analysing the variability between single plants, at different developmental stages and in subsequent generations, as opposed to only a pool of plants from a single mutant generation. Recently, the mechanism of rDNA repeat stabilisation during meiosis was revealed, as HR is specifically downregulated in the nucleolus and DNA breaks are repaired via NHEJ [85]. It would be of special interest to see whether there is an effect on rDNA repeat stability following the depletion of FANCJB and RTEL1, during meiosis.

When we originally discovered rDNA instability in the *rtel1 rmi2* double mutant background, we postulated that this phenotype goes along with a general instability of repeated sequences [30]. This was because both single, as well as the double mutant, showed a hyperrecombination phenotype for a repeated transgene marker. However, this is not the case for AtFANCJB, which as demonstrated by the use of the same marker (Fig 2D), is not involved in the suppression of homologous recombination. Therefore, we postulate that the helicases are required for the processing of complex replication intermediates. Due to its repetitive nature and sequence composition, the ribosomal DNA is especially prone to the formation of such complex secondary structures. G-quadruplexes (G4) are extremely stable secondary structures stabilized by Hoogsteen hydrogen bonding of guanine bases [86,87]. Analyses from *P*. *patens* also demonstrated the presence of a highly G4-prone sequence in the linker between 5S and 45S rDNA repeats [80]. For both FANCJ and RTEL1 helicases, functions in the unwinding of G4 DNA structures have been postulated, hinting now to a link between rDNA repeat stability and G4 DNA unwinding in Arabidopsis. The analyses of RTEL1 in G4 homeostasis mainly concentrated on telomeres where mammalian RTEL1 was postulated to unwind telomeric G4 structures [22,88]. But also a function for human RTEL1 in the suppression of trinucleotide repeat expansions, probably by the unwinding of hairpin structures, was postulated, allowing the possibility of a broader substrate spectrum [89]. FANCJ and its homologs from various organisms have been thoroughly described in respect to their role in G4 metabolism [12]. The ability of human FANCJ to unwind G4 DNA structures was shown to be independent of the FA pathway and an *in vivo* function could be demonstrated, as FANCJ depleted cell lines depicted hypersensitivity towards G4 ligands [13]. The identification of DOG-1 in *C*. *elegans* was even based on a mutant phenotype with deletions in genes containing polyguanine tracts, now known to be based on the function of the helicase in unwinding G4s [90,91]. Similar phenotypes were also reported for human cell lines, furthermore a function for mammalian FANCJ in the maintenance of microsatellites was also demonstrated [63,64,92]. For the human RTR complex helicase BLM, an G4 unwinding function *in vitro* has also been shown and recent studies hint to an *in vivo* role in the stabilisation of G4-rich sequences [93,94]. Based on the knowledge about the helicases FANCJ and RTEL1, as well as the RTR complex, it is now tempting to speculate if the effects of these factors we see on rDNA repeat stability in Arabidopsis is based on their function to unwind G4 structures. Interestingly, it was shown before that the non-transcribed spacer (NTS) sequence, naturally located between rDNA genes, enhances homologous recombination when positioned next to a recombination substrate [95]. This might be based on the specific blockage of the replication fork at a possible internal replication fork barrier (RFB). It could be that in the absence of FANCJB, a barrier for replication forks on G4-prone rDNA sequences is similarly induced, thereby increasing recombination at these sites, leading to a loss of rDNA copies, but not detectable in our artificial recombination marker lacking such G4-rich regions. It would be interesting to test this hypothesis by additional analyses with a modified reporter system. The helicases FANCJB and RTEL1 might complement each other in the unwinding of G4 DNA, but in the absence of both, the presence of persisting G4 structures might strongly hinder replication, leading to a dramatic loss of still non-replicated rDNA copies. As the absence of either helicase in humans is associated with a hereditary disease, Fanconi Anemia and Hoyeraal-Hreidarsson syndrome, respectively, it is of utmost interest to see if rDNA instability occurs in humans and has pathological consequences.

## Materials and methods

### Plant material and growth conditions

The *Arabidopsis thaliana* lines used in this study were in the Columbia (Col-0) background. For the characterization of FANCJA and FANCJB in Arabidopsis, the T-DNA insertion lines *fancja-1* (SALK_079991) and *fancjb-1* (SALK_101493), respectively, were used. Additional *fancja* mutant lines were generated using CRISPR/Cas9 mediated mutagenesis with a *S*. *pyogenes* Cas9 double nickase approach, as described before [42]. For the generation of *fancja* single mutants, mutagenesis was performed in Arabidopsis wild type plants, for *fancja fancjb* double mutants, mutagenesis was carried out in *fancjb-1* mutants. The *fancjb-2* and *fancjb-3* mutants were produced by *S*. *aureus* Cas9-mediated mutagenesis, as described in [43]. Further double mutants were generated by crossbreeding of *fancjb-1* with the previously described *fan1-1* (GABI_815C08), *recq4A-4* (GABI_203C07), *mus81-1* (GABI_113F11), *rad5A-2* (SALK_047150), *rev3-5* (SALK_126789) and *rtel1-1* (SALK_113285) mutant lines from the SALK and GABI-Kat collections, respectively [19, 21, 33, 35, 38, 40, 96, 97]. For cultivation in the greenhouse, plants were sown on a 1:1 mixture of Floraton 3 (Floragard, Oldenburg, Germany) and vermiculite (2–3 mm, Deutsche Vermiculite Dämmstoff, Sprockhövel, Germany) and grown at 22 °C with 16 h light and 8 h dark. The cultivation of plants for assays was performed under axenic conditions as described before [49].

### Fertility assays

To analyse fertility, Arabidopsis plants were grown in the greenhouse. Five mature siliques of five plants per line were harvested and transferred into 70% EtOH. After incubation overnight, the silique length and number of seeds per silique were determined using a binocular microscope.

### Sensitivity assays

Sensitivity analysis was performed as previously described [49]. Plants were grown in axenic culture for 7 days, and then 10 seedlings were transferred into each well of a six-well plate containing 5 ml GM medium for negative controls, or 4 ml for genotoxin treated samples. The following day, 1 ml genotoxin solution was added to obtain the intended concentration. After 13 days of incubation, the fresh weight of the plants was determined. The fresh weight of each treated sample was normalised to the fresh weight of the untreated control.

### HR assays

HR assays utilizing the IC9C reporter construct were performed as described before [33, 44]. Surface sterilized seeds were sown on GM medium and incubated for one week. 40 plantlets of each line were transferred into halved Petri dishes containing 10 ml liquid GM medium. After one week of incubation, plants were histochemically GUS stained as described. Then the blue sectors per plant were counted using a binocular microscope to determine the HR rate.

### Root growth assays

For the analysis of root growth, seeds were grown vertically under axenic conditions on square plates containing GM medium with 1% plant agar. For root length assays, plants were incubated for 10 days, pictures were taken and image analysis was performed using the ImageJ plugin SmartRoot [98]. For the analysis of cell death in the root meristem, plants were incubated for 5 days followed by propidium iodide staining as previously described [19].

### Quantitative Real Time PCR

For the quantification of 45S and 5S rDNA copy number, quantitative Real Time PCR was performed, as previously described [30]. Therefore, genomic DNA was isolated from 2-week-old plantlets grown in axenic culture. For qRT-PCR analysis, the LightCycler 480 KAPA SYBR Fast Mastermix (Sigma-Aldrich, Steinheim, Germany) was used in total reaction volumes of 10 μl (5 μl KAPA SYBR Fast Mastermix, 0.5 μl of each primer [10 μM], 0.5 ng gDNA, filled up with distilled water). The analysis was performed using a Light Cycler 480 (Roche Diagnostics, Mannheim, Germany). Data was normalized by amplification of the Ubiquitin 10 gene. For primer sequences see S3 Table.

### Fluorescence in situ hybridisation

The cytological quantification of 45S rDNA repeats via fluorescence in situ hybridisation (FISH) was performed as previously described [30]. Images were acquired with identical parameters and the area of 45S rDNA probe signals was quantified using ImageJ with background substraction using the “rolling ball” algorithm and a threshold of 16 standard deviations.

### Accession numbers

Sequence data from this article can be found with the following Arabidopsis AGI locus identifiers, AtFANCJA: At1g20750, AtFANCJB: At1g20720, AtFAN1: At1g48360, AtRECQ4A: At1g48360, AtMUS81: At4g30870, AtRAD5A: At5g22750, AtREV3: At1g67500, AtRTEL1: At1g79950.

## Supporting information

S1 Fig Characterization of T-DNA insertions from At*fancj* mutant lines. (A) The T-DNA in *fancja-1* is inserted in intron 24. (B) The T-DNA insertion in *fancjb-1* is positioned in intron 10 and accompanied by a 15 bp genomic deletion. Grey box: T-DNA with left borders (LB), red/green box: adjoining genomic sequences.
(PDF)

S2 Fig Analysis of genomic mutations in *fancja-2* and *fancja-3* mutant lines. Induced mutations in *fancja-2* (A) and *fancja-3* (B) were determined by sequencing of genomic DNA. Mutant line gDNA sequences were aligned with the wild type (WT) reference; sequences differing from the WT are depicted in red. Both mutant lines harbour complex mutations consisting of a combination of deletions, insertions and substitutions.
(PDF)

S3 Fig cDNA analysis of *fancja-2*, *fancja-3*, *fancja-4* and *fancja-5* mutant lines. Induced mutations in the *fancja* mutant lines were verified by sequencing of cDNA. The mutations in *fancja-4* and *fancja-5* were identical on gDNA and cDNA level. Mutant line cDNA sequences were aligned with the wild type (WT) reference; sequences differing from the WT are depicted in red. Premature stop codons in frame are featured in a red box. The mutations in all mutant lines lead to a frameshift in the open reading frame, resulting in a premature stop codon.
(PDF)

S4 Fig cDNA analysis of *fancjb-2* and *fancjb-3* mutant lines. Induced mutations in the *fancjb* mutant lines were verified by sequencing of cDNA. The induced mutations were identical on gDNA and cDNA level. Mutant line cDNA sequences were aligned with the wild type (WT) reference; sequences differing from the WT are depicted in red. Premature stop codons in frame are featured in a red box. The mutations in both mutant lines lead to a frameshift in the open reading frame, resulting in a premature stop codon.
(PDF)

S5 Fig Fertility analysis of *fancja-1* and *fancjb-1* mutant lines. Average silique length (A) and seeds per silique (B) were determined for *fancja-1* and *fancjb-1* mutant lines in comparison to wild type (WT) plants. Fertility of both mutant lines did not differ from the WT. Statistical differences were calculated using the two-tailed t-test with unequal variances: ns = not significant.
(PDF)

S1 Table Primer combinations for genotyping. (PDF)

S2 Table Primer sequences for genotyping. (PDF)

S3 Table Primer combinations for qRT-PCR. (PDF)
