# Supplementary figures and images for: An Arabidopsis FANCJ helicase homologue is required for DNA crosslink repair and rDNA repeat stability
Source: PLoS Genet. 2019 May 23;15(5):e1008174. doi: 10.1371/journal.pgen.1008174 (PMC6550410; doi:10.1371/journal.pgen.1008174)

**A** *fancja-1* T-DNA insertion mutant SALK\_079991

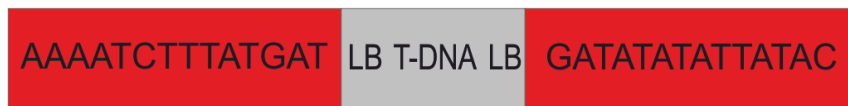

**B** *fancjb-1* T-DNA insertion mutant SALK\_101493

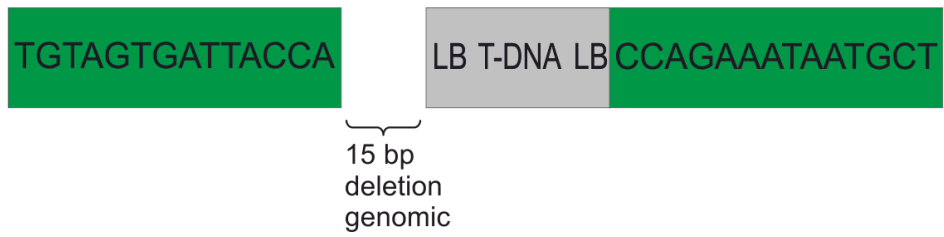

Supplement: S1 Fig — (A) The T-DNA in fancja-1 is inserted in intron 24. (B) The T-DNA insertion in fancjb-1 is positioned in intron 10 and accompanied by a 15 bp genomic deletion. Grey box: T-DNA with left borders (LB), red/green box: adjoining genomic sequences. (PDF) [file pgen.1008174.s001.pdf]

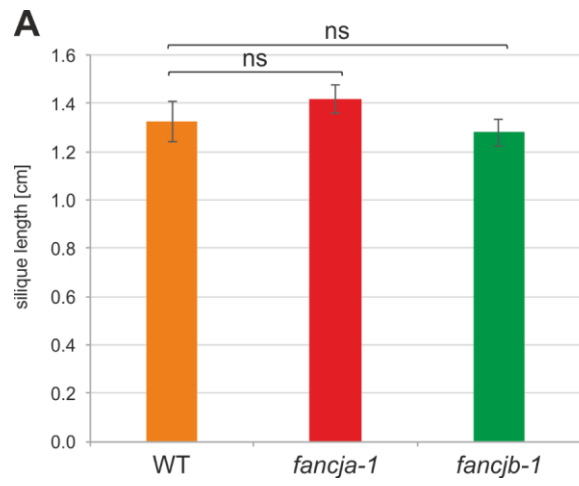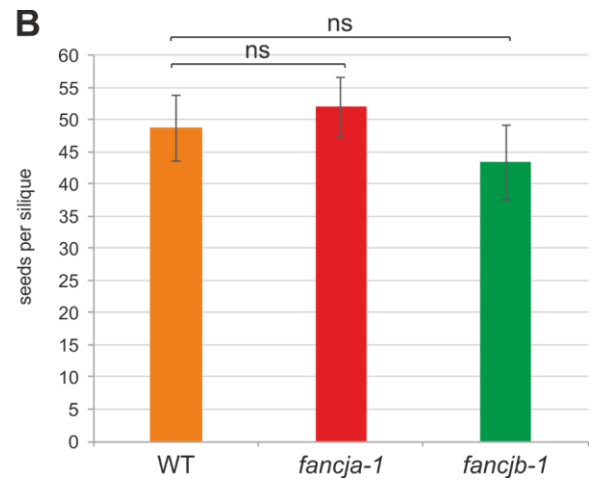

Supplement: S5 Fig — Average silique length (A) and seeds per silique (B) were determined for fancja-1 and fancjb-1 mutant lines in comparison to wild type (WT) plants. Fertility of both mutant lines did not differ from the WT. Statistical differences were calculated using the two-tailed t-test with unequal variances: ns = not significant. (PDF) [file pgen.1008174.s005.pdf]
