# Supplementary material for: An Arabidopsis FANCJ helicase homologue is required for DNA crosslink repair and rDNA repeat stability
Source: PLoS Genet. 2019 May 23;15(5):e1008174. doi: 10.1371/journal.pgen.1008174 (PMC6550410; doi:10.1371/journal.pgen.1008174)
Supplement: S2 Fig — Induced mutations in fancja-2 (A) and fancja-3 (B) were determined by sequencing of genomic DNA. Mutant line gDNA sequences were aligned with the wild type (WT) reference; sequences differing from the WT are depicted in red. Both mutant lines harbour complex mutations consisting of a combination of deletions, insertions and substitutions. (PDF) [file pgen.1008174.s002.pdf]

```
A   WT          3130 CCTAAACTGATGCATTCTTATTCTTGGTTGCAGAACCAAGAGGAGGATC-CAAGGACGATTTCGAAACTGTTCTCAAG---GAATACTATGAT 3218
    fancja-2         CCTAAACTGA---A--C-GATT-T------C-GAA--A-----CTGTTCTCAAGGACGATTTCGAAACTGTT---AAGAAAGCATACTATGAT

B   WT          3147 TTATTCTTGGTTGCAGAACCAAGA-GGA--GGATCCAAGGACGA-TTTCGAAACTGTTCT 3202
    fancja-3         TTATTCTTGG--AAAGAAC--AGATTGATTGG----AAGAAC-AGTTTCAAAACTGTTCT
```
