# Supplementary material for: An Arabidopsis FANCJ helicase homologue is required for DNA crosslink repair and rDNA repeat stability
Source: PLoS Genet. 2019 May 23;15(5):e1008174. doi: 10.1371/journal.pgen.1008174 (PMC6550410; doi:10.1371/journal.pgen.1008174)
Supplement: S3 Fig — Induced mutations in the fancja mutant lines were verified by sequencing of cDNA. The mutations in fancja-4 and fancja-5 were identical on gDNA and cDNA level. Mutant line cDNA sequences were aligned with the wild type (WT) reference; sequences differing from the WT are depicted in red. Premature stop codons in frame are featured in a red box. The mutations in all mutant lines lead to a frameshift in the open reading frame, resulting in a premature stop codon. (PDF) [file pgen.1008174.s003.pdf]

A

```
WT         1613 AACCAAGAGGAGGATCCAAGGACGATTTCGAAACTGTTCTCAAG---GAA 1659
fancja-2        --------------------GACGATTTCGAAACTGTT---AAGAAAGCA
                                                      STOP

WT         1660 TACTATGATTCGATAAGCGGAAAGAACAGATTGATTGGAAGAAATAGCAG 1709
fancja-2        TACTATGATTCGATAAGCGGAAAGAACAGATTGATTGGAAGAAATAGCAG

WT         1710 CGTTAAGAAAGCAGGGTCTGTTATAACTGAGGCTCAAGATGATTCCAAGA 1759
fancja-2        CGTTAAGAAAGCAGGGTCTGTTATAACTGAGGCTCAAGATGATTCCAAGA

WT         1760 GAGGATCTGCATTTCTTGCAGTATGTAGAGGAAAG
fancja-2        GAGGATCTGCATTTCTTGCAGTATGTAGAGGAAAG
```

B

```
WT         1613 AACCAAGA--GGAGGATCCAAGGACGATTTCGAAACTGTTCTCAAGGAAT 1660
fancja-3        A---TTGATTGGAAGAAC--AG-----TTTCGAAACTGTTCTCAAGGAAT
                    STOP

WT         1661 ACTATGATTCGATAAGCGGAAAGAACAGATTGATTGGAAGAAATAGCAGC 1710
fancja-3        ACTATGATTCGATAAGCGGAAAGAACAGATTGATTGGAAGAAATAGCAGC

WT         1711 GTTAAGAAAGCAGGGTCTGTTATAACTGAGGCTCAAGATGATTCCAAGAG 1760
fancja-3        GTTAAGAAAGCAGGGTCTGTTATAACTGAGGCTCAAGATGATTCCAAGAG

WT         1761 AGGATCTGCATTTCTTGCAGTATGTAGAGGAAAG
fancja-3        AGGATCTGCATTTCTTGCAGTATGTAGAGGAAAG
```

C

```
WT         1613 AACCAAGA-------------------------GGAGGATCCAAGGACGA 1637
fancja-4        AACCAAGAGGAATTCTTGGTTGCAGAACCAAGAGGAGGATCCAAGGACGA

WT         1638 TTTCGAAACTGTTCTCAAGGAATACTATGATTCGATAAGCGGAAAGAACA 1787
fancja-4        TTTCGAAACTGTTCTCAAGGAATACTATGATTCGATAAGCGGAAAGAACA
                                            STOP

WT         1688 GATTGATTGGAAGAAATAGCAGCGTTAAGAAAGCAGGGTCTGTTATAACT 1737
fancja-4        GATTGATTGGAAGAAATAGCAGCGTTAAGAAAGCAGGGTCTGTTATAACT

WT         1738 GAGGCTCAAGATGATTCCAAGAGAGGATCTGCATTTCTTGCAGTATGTAG 1787
fancja-4        GAGGCTCAAGATGATTCCAAGAGAGGATCTGCATTTCTTGCAGTATGTAG

WT         1788 AGGAAAG
fancja-4        AGGAAAG
```

D

```
WT         1613 AACCAAGA-----------------------------------GGAGGAT 1627
fancja-5        AACCAAGAGGAGGATATTCTTATTCTTGGTTGCAGAACCAAGAGGAGGAT

WT         1628 CCAAGGACGATTTCGAAACTGTTCTCAAGGAATACTATGATTCGATAAGC 1677
fancja-5        CCAAGGACGATTTCGAAACTGTTCTCAAGGAATACTATGATTCGATAAGC
                                                              STOP

WT         1678 GGAAAGAACAGATTGATTGGAAGAAATAGCAGCGTTAAGAAAGCAGGGTC 1727
fancja-5        GGAAAGAACAGATTGATTGGAAGAAATAGCAGCGTTAAGAAAGCAGGGTC

WT         1728 TGTTATAACTGAGGCTCAAGATGATTCCAAGAGAGGATCTGCATTTCTTG 1777
fancja-5        TGTTATAACTGAGGCTCAAGATGATTCCAAGAGAGGATCTGCATTTCTTG

WT         1778 CAGTATGTAGAGGAAAG
fancja-5        CAGTATGTAGAGGAAAG
```
