# Supplementary material for: An Arabidopsis FANCJ helicase homologue is required for DNA crosslink repair and rDNA repeat stability
Source: PLoS Genet. 2019 May 23;15(5):e1008174. doi: 10.1371/journal.pgen.1008174 (PMC6550410; doi:10.1371/journal.pgen.1008174)
Supplement: S4 Fig — Induced mutations in the fancjb mutant lines were verified by sequencing of cDNA. The induced mutations were identical on gDNA and cDNA level. Mutant line cDNA sequences were aligned with the wild type (WT) reference; sequences differing from the WT are depicted in red. Premature stop codons in frame are featured in a red box. The mutations in both mutant lines lead to a frameshift in the open reading frame, resulting in a premature stop codon. (PDF) [file pgen.1008174.s004.pdf]

A

```
WT            1564 GTGTGGCGGGTGCAATCTCCAATGGTCCTAGTAATTATCCTCTAAATGC 1613
fancjb-2           GTG----------CAATCTCCAATGGTCCTAGTAATTATCCTCTAAATGC
                                                             STOP

WT            1614 AAGTTATAAAACAGCTGATGCATATTCATTCCAG
fancjb-2           AAGTTATAAAACAGCTGATGCATATTCATTCCAG
```

B

```
WT            1691 AATTACAGAATGAACTAGAACAGATGAGCGTGGCGCAGCCAATGATTTAT 1740
fancjb-3           AATTACAGAATGAACTAGAACAGATGAGCGTGGCGCAGCCAATGA-----

WT            1641 CAACCCTTGTGTGAAGTAGTAGAG
fancjb-3           --------------AGTAGTAGAG
                                   STOP
```
