# Supplementary material for: An Arabidopsis FANCJ helicase homologue is required for DNA crosslink repair and rDNA repeat stability
Source: PLoS Genet. 2019 May 23;15(5):e1008174. doi: 10.1371/journal.pgen.1008174 (PMC6550410; doi:10.1371/journal.pgen.1008174)
Supplement: S1 Table — (PDF) [file pgen.1008174.s006.pdf]

S1 Table: Primer combinations for genotyping.

Line	Primer combination	Product length	Anneal. temp.
fancja-1	FANCJA-4686-4706 / FANCJA-5674-5654	1000 bp	58 °C
	FANCJA-4686-4706 / SALK LB1	1000 bp	58 °C
fancjb-1	fancjB-1 FW1 / fancjB-1 RV1	2000 bp	58 °C
	fancjB-1 RV1 / LBb1.3	900 bp	58 °C
fan1-1	FAN1-2 / FAN1-R2	600 bp	56 °C
	FAN1-2 / LB1	600 bp	56 °C
recq4A-4	RQ4A-(-2A) / RQ4A-R6A	1000 bp	56 °C
	LB1 / RQ4A-R6A	500 bp	56 °C
mus81-1	MUS81-1 / MUS?-R2	600 bp	55 °C
	LB1 / MUS?-R2	500 bp	56 °C
rad5A-2	rad5A fw / rad5A rv	1100 bp	56 °C
	rad5A rv / LBb1.3	600 bp	54 °C
rev3-5	SK-75 / SK-76	1000 bp	56 °C
	SK-75 / Lbd1	1000 bp	56 °C
rtel1-1	RTEL1-IN5-FW2 / RTEL1-IN7-RW2	700 bp	56 °C
	RTEL1-IN5-FW2 / LBd1	750 bp	56 °C
