# Supplementary material for: An Arabidopsis FANCJ helicase homologue is required for DNA crosslink repair and rDNA repeat stability
Source: PLoS Genet. 2019 May 23;15(5):e1008174. doi: 10.1371/journal.pgen.1008174 (PMC6550410; doi:10.1371/journal.pgen.1008174)
Supplement: S2 Table — (PDF) [file pgen.1008174.s007.pdf]

S2 Table: Primer sequences for genotyping.

Primer name	Sequence (5‘-3‘)
**FANCJA-4686-4706**	GAATCGTCTGTAACTCCTGAG
**FANCJA-5674-5654**	GTTTCCCGATCTGTACCACTG
**SALK LB1**	TGGTTCACGTAGTGGGCCATCG
**fancjB-1 FW1**	CTTGGGAAAGACAATGTAGATGATG
**fancjB-1 RV1**	GTATGCGTCCAGGTACCAG
**LBb1.3**	ATTTTGCCGATTTCGGAAC
**FAN1-2**	GCAAAGGCGGATTCTTCG
**FAN1-R2**	GAAGCAGGTCTTACTTTGC
**rad5A fw**	GCGTCTTCGACAATGTTGTG
**rad5A rv**	CAGGATACTGCCATCTTCGG
**SK-75**	GCCCTGAAGCCTTCCTTACTTG
**SK-76**	GAAGAGGCTAGTTCAAACGTCC
**Lbd1**	TCGGAACCACCATCAAACAG
**RTEL1-IN5-FW2**	AGGGACTAATGTTGTTTCAC
**RTEL1-IN7-RW2**	GGACGAATGAGATGGTGTA
**LB1**	GACCATCATACTCATTGCTG
**MUS?-R2**	GCAGCATCAATAAGCTCTTG
**MUS81-1**	AGTGAATCTGATAGTGAGTG
**RQ4A-(-2A)**	GTCCTGATCGTGTTGGACAG
**RQ4A-R6A**	ATCAGAGCCACTCATTGTTG
