# Supplementary material for: An Arabidopsis FANCJ helicase homologue is required for DNA crosslink repair and rDNA repeat stability
Source: PLoS Genet. 2019 May 23;15(5):e1008174. doi: 10.1371/journal.pgen.1008174 (PMC6550410; doi:10.1371/journal.pgen.1008174)
Supplement: S3 Table — (PDF) [file pgen.1008174.s008.pdf]

S3 Table: Primer combinations for qRT-PCR.

Target	Primer name	Sequence (5‘-3‘)
Ubiquitin 10	UBQ10 F	AACGGGAAAGACGATTAC
	UBQ10 R	ACAAGATGAAGGGTGGAC
**18S rDNA**	18S (XbaI) F	CTAGAGCTAATACGTGCAACAAAC
	18S (HpaI) R	GAATCGAACCCTAATTCTCCG
**5.8S rDNA**	5,8S FW	CGGAGTGTGGGCGGATG
	5,8S RV	GTGAGGGACGACGATTTG
**25S rDNA**	25S FW	GTGCGAGTCAACGGGTG
	25S RV	ACCCAAGTCAGACGAACG
**5S rDNA**	FW1_qPCR_5SrDNA	TAACGGATGCGATCATACCAG
	RV1_qPCR_5SrDNA	AGGTATCACATGCCAAGTTTGG
